# Luminal and Glandular Epithelial Cells from the Porcine Endometrium maintain Cell Type-Specific Marker Gene Expression in Air–Liquid Interface Culture

**DOI:** 10.1007/s12015-022-10410-3

**Published:** 2022-07-18

**Authors:** Meret Schmidhauser, Susanne E. Ulbrich, Jennifer Schoen

**Affiliations:** 1 Animal Physiology, Institute of Agricultural Sciences, ETH Zurich, Zurich, Switzerland; 2 Institute of Reproductive Biology, Research Institute for Farm Animal Biology (FBN), Dummerstorf, Germany; 3 Reproduction Biology Department, Leibniz Institute for Zoo and Wildlife Research IZW, Berlin, Germany

**Keywords:** Endometrium, Glandular epithelium, Luminal epithelium, Air–liquid interface, Cell culture

## Abstract

Two different types of epithelial cells constitute the inner surface of the endometrium. While luminal epithelial cells line the uterine cavity and build the embryo-maternal contact zone, glandular epithelial cells form tubular glands reaching deeply into the endometrial stroma. To facilitate investigations considering the functional and molecular differences between the two populations of epithelial cells and their contribution to reproductive processes, we aimed at establishing differentiated *in vitro* models of both the luminal and the glandular epithelium of the porcine endometrium using an air–liquid interface (ALI) approach. We first tested if porcine luminal endometrium epithelial cells (PEEC-L) reproducibly form differentiated epithelial monolayers under ALI conditions by monitoring the morphology and the trans-epithelial electrical resistance (TEER). Subsequently, luminal (PEEC-L) and glandular epithelial cells (PEEC-G) were consecutively isolated from the endometrium of the uterine horn. Both cell types were characterized by marker gene expression analysis immediately after isolation. Cells were separately grown at the ALI and assessed by means of histomorphometry, TEER, and marker gene expression after 3 weeks of culture. PEEC-L and PEEC-G formed polarized monolayers of differentiated epithelial cells with a moderate TEER and *in vivo*-like morphology at the ALI. They exhibited distinct patterns of functional and cell type-specific marker gene expression after isolation and largely maintained these patterns during the culture period. The here presented cell culture procedure for PEEC-L and -G offers new opportunities to study the impact of embryonic signals, endocrine effectors, and reproductive toxins on both porcine endometrial epithelial cell types under standardized *in vitro* conditions.

**Graphical Abstract:**

Created with BioRender.com.

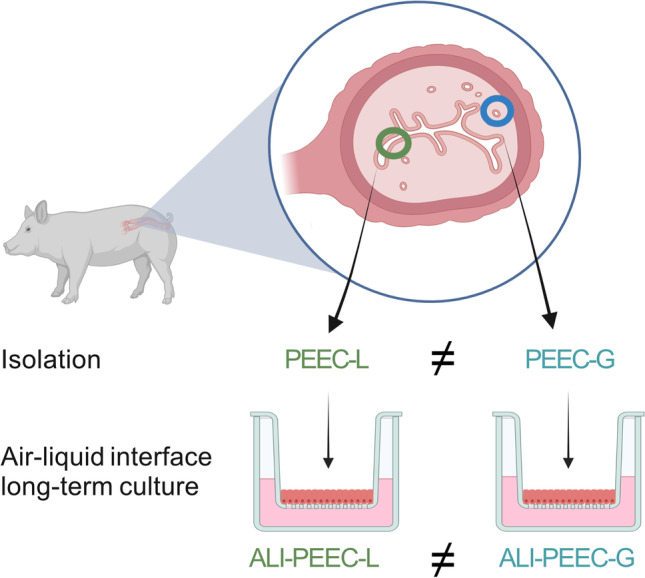

## Introduction

The endometrium, the inner mucosal layer of the uterine cavity, is lined with epithelial cells which shape the microenvironment of the conceptus on the one hand and at the same time form a barrier against immunological stimuli [1]. The endometrial epithelium can be divided into two distinct moieties, the luminal epithelium (LE) forming the surface of the uterine cavity, and the glandular epithelium (GE) which makes up the tubular glands and reaches deeply into the endometrial stroma. LE and GE differ regarding their development, localization, and function within the endometrium. The GE develops postnatally from the LE by bud formation, tubulogenesis, and later extensive branching [2]. While LE cells directly get in contact with the conceptus, uterine glands contribute to fertility and pregnancy success through their secretions which ensure conceptus survival, implantation, and placenta formation [3, 4]. In accordance with these divergent functions, the two cell types exhibit distinct molecular signatures [5]. Although many genes are expressed in both LE and GE, the expression patterns of genes driving uterine fluid secretion and embryo-maternal communication are characteristic for each cell type [6–9].

The individual physiology of endometrial LE and GE cells, their hormonal regulation, and contribution to conceptus support and uterine dysfunctions are difficult to investigate *in vivo*, both for technical and ethical reasons. Well-characterized *in vitro* models faithfully representing the distinct epithelial cell types are needed to fill this gap.

Cultivation under standard 2D adherent submersed conditions affects the differentiation and functionality of cells. This is especially true for epithelial cells, whose barrier and selective transport functions directly depend on their baso-apical polarization. Alternative approaches that foster *in vivo*-like differentiation of the epithelium *in vitro* are 3D organoid cultures using extracellular matrix components or compartmentalized culture systems based on porous filter supports. Both methods allow *in vivo*-like nutrition of the cells from the basolateral cell side and are commonly used to establish highly differentiated epithelial *in vitro* models. Organoid cultures can be established from a very low number of input cells as they have self-organizing properties, self-renewal capacity and host differentiated as well as progenitor cells [10–13]. Therefore, it is the method of choice if the cell source is scarce (e.g. cells from biopsy or other small samples). However, in an organoid, the apical (luminal) cell side is experimentally accessible only by puncturing the cell layer, which renders the application of apical effectors (such as embryonic signals in the case of the endometrium) and the repeated analysis of apical secretions difficult without affecting the cells. If the starting material to obtain primary cells is not a limiting factor (as in farm animals bred for food production), compartmentalized culture systems are a straightforward option for generating differentiated epithelial *in vitro* systems [14]. Especially culture at the air–liquid interface (ALI), where the apical cell side is exposed to ambient air and kept free of growth medium, supports excellent baso-apical epithelium polarization, barrier formation, and long-term cell survival [15–18].

To establish an ALI culture, cells are first grown with medium supply from both the apical and baso-lateral cell side (submerged pre-culture) to allow formation of a confluent epithelial monolayer. Subsequently, the apical medium is removed to stimulate baso-apical polarization and differentiation. The medium in the basal compartment is either the same during submerged and ALI culture (one-step approach) or is changed between the two culture periods allowing application of a medium predominantly supporting proliferation in the submerged pre-culture and a differentiation medium during ALI culture (two-step approach) [19].

ALI culture systems of human and bovine endometrial epithelial cells have successfully been established [20, 21]. Despite their different localization within the endometrium and the different contributions of LE and GE to endometrial functions, to the best of our knowledge no protocol has yet been presented for the separate isolation and cultivation of the two different epithelial types.

We therefore aimed at establishing ALI culture procedures for both LE and GE cells of the porcine endometrium (PEEC-L and PEEC-G, respectively). We focused on the porcine endometrium as the pig is an important farm animal as well as a widely used biomedical model species. Furthermore, samples for cell collection are readily available in large quantities from slaughterhouse by-products. We tested the hypotheses that i) primary cells of both the LE and GE form differentiated epithelial monolayers under ALI conditions and ii) both epithelial types maintain their cell type specific differences after ALI culture. For this purpose, we first verified the general feasibility of the ALI approach for PEEC-L and tested different culture conditions. In a second step, both PEEC-L and -G were isolated to establish ALI-PEEC-L and ALI-PEEC-G. These were then comparatively characterized regarding morphology, barrier function and marker gene expression.

## Materials and Methods

### Media and Reagents

Media compositions applied for cell culture purposes are listed in Table 1. Phenol red-free DMEM/ Hams F12 1:1 (21,041,025), Ham’s F12 (11,765,054), GlutaMAX (35,050,087), and Nu-serum growth medium supplement (11,563,600) were obtained from ThermoFisher Scientific, Waltham, USA. FBS (S0115, Lot 0742C), penicillin/streptomycin (A2212), amphotericin B (A2612), HEPES (L1613), and PBS (L1825), were purchased from Biochrom AG, Berlin, Germany. BSA (A9418), insulin (I6634), transferrin (T8158), cholera toxin (C8052), epidermal growth factor (E4127), bovine pituitary extract (P1476), ascorbic acid (A4544), retionic acid (R2625), collagen (C5533) and collagenase (C2674) were products of Sigma-Aldrich, St. Lous, USA. Chemicals for histological procedures were obtained from Carl Roth GmbH, Karlsruhe, Germany.

**Table 1:** Composition of media used for ALI-PEEC-L culture procedures

PBS supplemented with antibiotics	PBS supplemented with 100 U/mL penicillin / 100 μg/mL streptomycin and 1 μg/mL amphotericin B
Basic medium (B)	Phenol red-free DMEM/ Hams F12 1:1 supplemented with 100 U/mL penicillin / 100 μg/mL streptomycin, 1 μg/mL amphotericin B and 15 mM HEPES
Proliferation medium (P)	Medium B supplemented with 5% FBS, 10 μg/mL insulin, 5 μg/mL transferrin, 0.1 μg/mL cholera toxin, 25 ng/mL epidermal growth factor, 15 mg/mL bovine pituitary extract and 10 μg/mL retinoic acid
Differentiation medium containing Nu-Serum (NU)	Medium B supplemented with 3% FBS, 2% Nu-Serum growth medium supplement and 10 μg/mL retinoic acid
Differentiation medium serum- free (SF)	Medium B supplemented with BSA 1 mg/ml, 5 μg/mL insulin, 5 μg/mL transferrin, 0. 25 μg/mL cholera toxin, 2 ng/mL epidermal growth factor, 15 mg/mL bovine pituitary extract and 10 μg/mL retionic acid
One-step medium unconditioned (UM)	Ham’s F12 supplemented with 10% FBS, 100 U/mL penicillin / 100 μg/mL streptomycin, 1 μg/mL amphotericin B, 10 μg/mL ascorbic acid and 1 mM GlutaMAX
One-step medium conditioned (CM)	UM supplemented with 1/3 3T3 mouse fibroblast conditioned medium [22]

### Experiment 1: ALI Culture of PEEC-L and Media Comparison

In experiment 1, we verified if PEEC-L form differentiated epithelial monolayers under ALI conditions. The compositions of all applied media are provided in Table 1. We initially tested a two-step approach consisting of a submerged pre-culture period in a medium supporting proliferation (P), followed by differentiation at the ALI using a simple differentiation medium (NU) as previously reported for oviduct epithelial cells [23]. To monitor the development of the ALI culture, their morphology and barrier function (trans-epithelial electrical resistance, TEER) was assessed after 1, 2 and 3 weeks of culture (n = 5 biological replicates of primary cells from different donor animals). We furthermore tested if serum-free differentiation medium (SF) [23] or simple one-step media [unconditioned (UM) and conditioned media (CM), respectively] were applicable to culture PEEC-L. The morphology and barrier function of ALI-PEEC-L grown in different culture conditions was evaluated after three weeks of culture (n = 3–4 biological replicates of primary cells from different donor animals).

#### Tissue Collection and PEEC-L Isolation

Porcine uteri of healthy, 6-month-old gilts were collected from the local, institute-owned slaughterhouse. All animals were slaughtered for meat production purposes. Exclusively uteri of peri-pubertal, non-cycling animals with ovaries containing only small follicles and without any corpora lutea were used for cell isolation. Uteri were transported on ice and processed within 1 h after slaughter. The uteri were briefly washed with PBS supplemented with antibiotics (Table 1), and briefly disinfected with 70% Ethanol. Each uterine horn was then flushed three times with 20 mL of medium B (Table 1).

The uterine horns were closed with clamps, filled with 20 mL of collagenase type 1A solution (1 mg/ml in medium B) and incubated at 37 °C for 1 h on a shaker. The enzyme solution was collected in a tube containing medium B supplemented with 10% FBS. The horn was opened longitudinally, and PEEC-L were scraped off from the inner surface of the endometrium using a sterile glass slide. The remaining tissue was either discarded or used for further PEEC-G isolation (Experiment 2). PEEC-L were centrifuged at 200 g for 8 min, resuspended in medium B and centrifuged again. For detaching cell clusters, the washed cell pellet was resuspended in Accutase (Pan Biotech, P10-21,100) and incubated at 37 °C for 20 min. The digestion was stopped by adding medium B supplemented with 10% FBS. PEEC-L were centrifuged at 200 g for 8 min and resuspended in medium B. To obtain single cells, the suspension was filtered through a 40 µm sieve. PEEC-L were centrifuged again and resuspended in 1 ml medium (P, UM or CM). PEEC-L were counted and immediately seeded at a density of 1.5 × 10^5^ cells per 24-well insert with 0.4 μm pore size (Sarstedt, 83.3932.041) coated with human collagen type IV as previously described [24].

#### ALI-PEEC-L Cell Culture

Freshly seeded cells were grown in medium P, UM and CM, respectively, with 1 mL of medium on the basolateral side and 200 μl of medium on the apical side. After 1 week of culture, the ALI was introduced by removing the medium on the apical side of the insert and only supplying the basolateral side with 1 mL of medium. In the two-step approaches, cells grown in medium P in liquid–liquid mode were supplemented with medium NU or SF during ALI culture. In the one-step approach, cells grown in medium UM or CM were cultured with the same medium in both culture phases. Cells were maintained in humified atmosphere with 5% CO_2_, 5% O_2_ at 37 °C. Medium was changed twice per week.

#### Barrier Function Assessment

Before harvesting the cell cultures, the TEER was determined by an EVOM2 Epithelial Voltohmeter with STX2 electrodes (WPI, Sarasota, FL, USA). To minimize any potential offset, the electrodes were equilibrated in the medium for at least 1 h. The apical compartment was supplemented with 200 µL of medium. Before and after measuring the samples, a blank sample (cell culture insert without cells) was assessed. The final unit area resistance (Ω*cm^2^) was calculated by subtracting the blank value and normalization to the area (1/3 cm^2^) of the 24-well insert.

#### Morphology Assessment

All histological procedures followed the description of Chen et al. [25]. Paraplast embedded samples were cut into 3 µm thick sections for hematoxylin–eosin (HE) staining. HE stained images (5 × images/sample) were taken at 40 × magnification to measure the cellular height (5 × positions/image), count total cell number, and the number of secretory cells using the ImageJ software (National Institutes of Health, Bethesda, MD, USA) [26].

### Experiment 2: Isolation and ALI Cultivation of both PEEC-L and PEEC-G

In experiment 2, we isolated both PEEC-L and -G from the porcine endometrium and compared ALI-PEEC-L and ALI-PEEC-G (n = 4 biological replicates of primary cells from different donor animals) using the culture conditions tested in experiment 1. Samples for histological analyses were taken after 3 weeks of culture. Marker gene expression to verify cell type specific expression patterns was employed immediately after cell isolation and after 3 weeks of culture. The cell isolation of the PEEC-L, TEER measurement, histology and morphometry were conducted as described for experiment 1.

#### Tissue Collection and PEEC-G Isolation

PEEC-L were enzymatically dissociated, scraped off and isolated as described in experiment 1. Subsequently, PEEC-G were isolated from the same area of the uterine horn. The mucosal layer was separated with dissecting scissors from the submucosal muscle layer. The dissected tissue was further chopped manually and washed with medium B on a 100 µm cell strainer to remove blood, single cells and cell debris. Washed tissue pieces were incubated in collagenase type 1A solution (1 mg/ml in medium B) for 90 min at 37 °C on a shaker. Cell clusters were loosened up and separated from larger tissue pieces by gentle pipetting. To remove remaining larger pieces of tissue, the solution was filtered through sterile medical gauze. The cell suspension was centrifuged at 200 × g for 8 min. Tubular cell clusters were washed and collected with a 40 µm cell strainer, transferred to a fresh tube and centrifuged. The cell clusters were treated with Accutase to obtain a single-cell suspension and then seeded as described in experiment 1.

#### ALI-PEEC-L and -G Cell Culture

In experiment 2, the same culture conditions were applied as in experiment 1. All cell cultures were carried out with medium P for submerged pre-culture and with medium NU for differentiation at the ALI.

#### RNA Isolation

Total RNA from frozen cell culture samples was isolated using AllPrep DNA/RNA Mini Kit (Qiagen, Hilden, Germany, 80,284). The samples were lysed in 350 µl of RLT Plus buffer, and total RNA was isolated by adding 350 µl of 100% ethanol to the DNA spin column flow-through. RNA was eluted in 30 µl of RNase-free water. RNA integrity and concentration were determined with the Agilent 2100 Bioanalyzer RNA 6000 Nano kit (Agilent, Santa Clara, USA; 5067–1511). The samples displayed a RNA integrity number of 9.98 ± 0.08 (mean ± SD).

#### Gene Expression Analysis

In accordance with previous reports, Stanniocalcin-1 (*STC1*), Insulin like growth factor binding protein 2 (*IGFBP2*), and Angiopoietin-related protein 1 (*ANGPTL1*) were chosen as markers for PEEC-L while Wnt inhibitory factor 1 (*WIF1*), Forkhead box A2 (*FOXA2*) and Follistatin (*FST*) were employed as markers for PEEC-G [8, 27–31]. As functional markers for both types of endometrial epithelium we selected Estrogen receptor 1 (*ESR1*), Progesterone receptor (*PGR*), Mucin 1 (*MUC1*) and Mucin 16 (*MUC16*) [32–34].

Total RNA (200 ng from each insert) was used for cDNA synthesis with the RevertAid First Strand cDNA Synthesis Kit (ThermoFisher Scientific, K1621). The reaction mix per sample included 11 μl RNA in H_2_O, 0.5 μl Oligo(dT)_15_ primer, 0.5 μl random primer, 4 μl reaction buffer, 2 μl dNTPs, 1 μl RiboLock RNase Inhibitor and 1 μl reverse transcriptase. Incubation of the reaction mix was performed in a PCR cycler (25 °C for 5 min, 42 °C for 60 min, 70 °C for 5 min).

Quantitative real-time PCR (qPCR) was carried out using the KAPA SYBR FAST qPCR Kit (Kapa Biosystems, Wilmington, USA) on a CFX384 Real-Time PCR Detection System (Bio-Rad, Munich, Germany). The relative expression level (ΔCq) of each gene was generated by scaling the target gene Cq of each individual sample to the geometric mean of the Cq of three reference genes [H3 histone family member 3A (H3F3A), tyrosine 3-monooxygenase/tryptophan 5-monooxygenase activation protein zeta (*YWHAZ*) and beta-actin (*ACTB*)] as described previously by Chiumia et al. [35]. To calculate ΔΔCq to visualize the RNA expression pattern, ΔCq values of PEEC-G or ALI-PEEC-G were subtracted from ΔCq values of PEEC-L or ALI-PEEC-L, respectively. The sequences of commercially synthesized primers (Microsynth, Balgach, Switzerland) applied are listed in Table 2.

**Table 2:** Primers used for RT-qPCR

Gene	forward	reverse	length	accession number
YWHAZ	AGGCTGAGCGATATGATGAC	GACCCTCCAAGATGACCTAC	141	NM_001315726.1
ACTB	GATGACTCAGATCATGTTCGAGAC	CAGAGTCCATGACAATGCCA	113	XM_003124280.5
H3F3A	ACTGGCTACAAAAGCCGCTC	ACTTGCCTCCTGCAAAGCAC	223	NM_213930.1
ESR1	AGGGAAGCTCCTGTTTGCTCC	CGGTGGATATGGTCCTTCTCT	234	NM_214220.1
PGR	TGAGAGCACTAGATGCCGTTGCT	AGAACTCGAAGTGTCGGGTTTGGT	197	NM_001166488.1
MUC16	AGTGGCTATGCACCCCAGAC	ACCAGGCAGGAGCGGAATAC	191	XM_021085118.1
MUC1	AGCTGATTCTGGCCTTCCAAGACA	TGGTCAGGTTATAGGTGCCTGCTT	96	XM_021089730.1
STC1	GTCAAAGAGAGTTTAAAGTGCATCG	ACGTTTTCTGTTGAAGTCAGCTC	272	NM_001103212.1
ANGPTL1	GTTATCCCAGAGATTTAATGCCC	CAATCTTTGAATGGTCCTTCGT	109	NM_001109947.1
WIF1	GATGCTCACCAGGCAAGAGT	TCATAGAAGTATTCGGCCCGC	185	NM_001315718.1
FST	AAAACCTACCGCAACGAATG	CAGAAAACATCCCGACAGGT	110	NM_001003662.1
FOXA2	ATAAGGAGGGCAAGGGAAAA	AGTCAAAATTCGCAGGTGCT	110	XM_005672754.3
IGFBP2	CCTGTACTCCTTGCACATCC	AGAGACATCTTGCACTGTTTGAG	72	NM_214003.1

### Statistical Analyses

The data obtained from the gene expression analysis and morphometry was statistically analyzed using paired t-test, paired one-way ANOVA with Tukey`s multicomparison test or regression analysis. The regression analysis and the paired t-test was conducted in R (version 4.1.0) and the ANOVA was conducted in GraphPad Prism (version 8.2.0).

## Results

### PEEC-L at the ALI

**Fig. 1:**
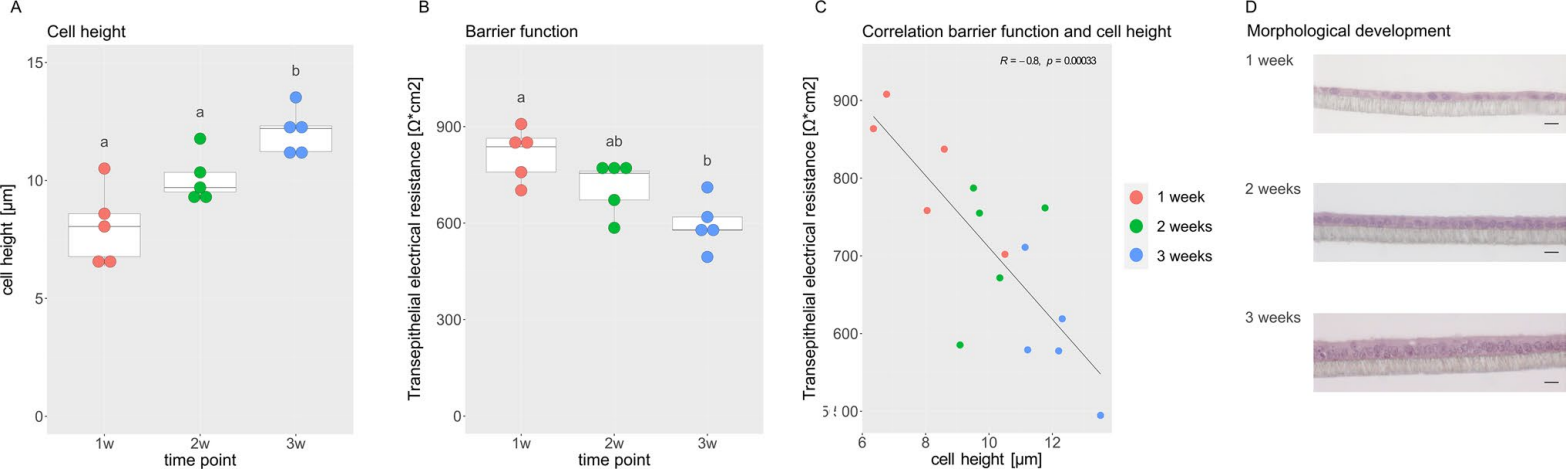
ALI-PEEC-L morphology and barrier function in long-term culture. (**A**) Cell height of PEEC-L after 1, 2 and 3 weeks of culture. (**B**) TEER of PEEC-L cells after 1, 2 and 3 weeks of culture. (**A**, **B**) The data are presented as a boxplot. Significant differences (*p* ≤ 0.05) between the time points are indicated by different superscript letters. (**C**) Correlation of cell height and TEER. The correlation was considered significant at *p* < 0.05. (**D**) Representative cross-sections of ALI-PEEC-L at 1, 2 and 3 weeks of culture, HE staining, magnification × 40, scale bar = 10 µm. *n* = 5. TEER, transepithelial electrical resistance; ALI, air–liquid interface; PEEC-L, porcine endometrial epithelial cells luminal; HE, hematoxylin–eosin

Isolated PEEC-L were cultured at the ALI for up to 3 weeks. Epithelial differentiation was confirmed by evaluating the morphology (cell height, columnar shape) and barrier function (TEER, Fig. 1). The height of the PEEC-L monolayer significantly increased during ALI culture (Fig. 1A) and reached 12.07 ± 0.38 µm (mean ± SEM). TEER showed an inverse pattern and decreased significantly between weeks 1 and 3 (Fig. 1B). The TEER was 814 ± 33 (mean ± SEM) after 1 week and 596 ± 32 (mean ± SEM) after 3 weeks. The cell height and TEER displayed a significant negative correlation (*p* ≤ 0.05) (Fig. 1C). Representative pictures of HE stained cells from one single animal illustrate the increase of cell height and gradual polarization over the culture period of 3 weeks (Fig. 1D).

### Applicability of Different Media for PEEC-L Culture at the ALI

**Fig. 2:**
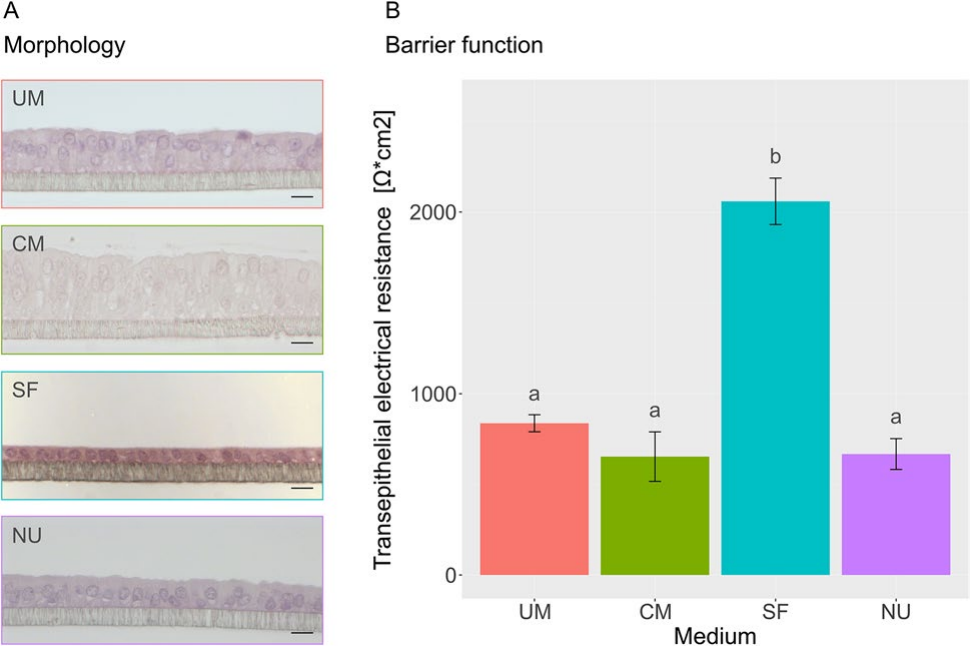
Morphology and barrier function of ALI-PEEC-L depend on medium composition. (**A**) Representative cross-sections of PEEC-L at the ALI after 3 weeks of culture in UM, CM, SF and NU, HE staining, magnification × 40, scale bar = 10 µm. (**B**) Transepithelial electrical resistance of ALI-PEEC-L after 3 weeks of culture. The results are presented as mean ± SEM. Significant differences (*p* ≤ 0.05) between the media are indicated by different superscript letters, *n* = 3–4. TEER, transepithelial electrical resistance; ALI, air–liquid interface; PEEC-L, porcine endometrial epithelial cells luminal; UM, unconditioned medium; CM, conditioned medium; SF, serum free medium; NU Nu-Serum medium; HE, hematoxylin–eosin

Three additional media were tested for their applicability to support differentiation of PEEC-L at the ALI (Fig. 2). After 3 weeks of culture, the media were evaluated based on TEER, cell morphology and homogeneity of the cell monolayer. Cells formed homogenous monolayers in all four media. However, the cells grown in SF medium displayed a cuboidal shape, while the cells in NU, UM or CM showed *in vivo*-like columnar shape. Additionally, the mean TEER of SF cultures was significantly higher than the mean TEER of the cells grown in other media. Therefore, it was concluded, that NU medium, CM and UM are generally suitable media for culturing PEEC-L at the ALI. Because of its low FBS content, we performed the following experiments with NU medium.

### Isolation of PEEC-L and PEEC-G from One Uterus

**Fig. 3:**
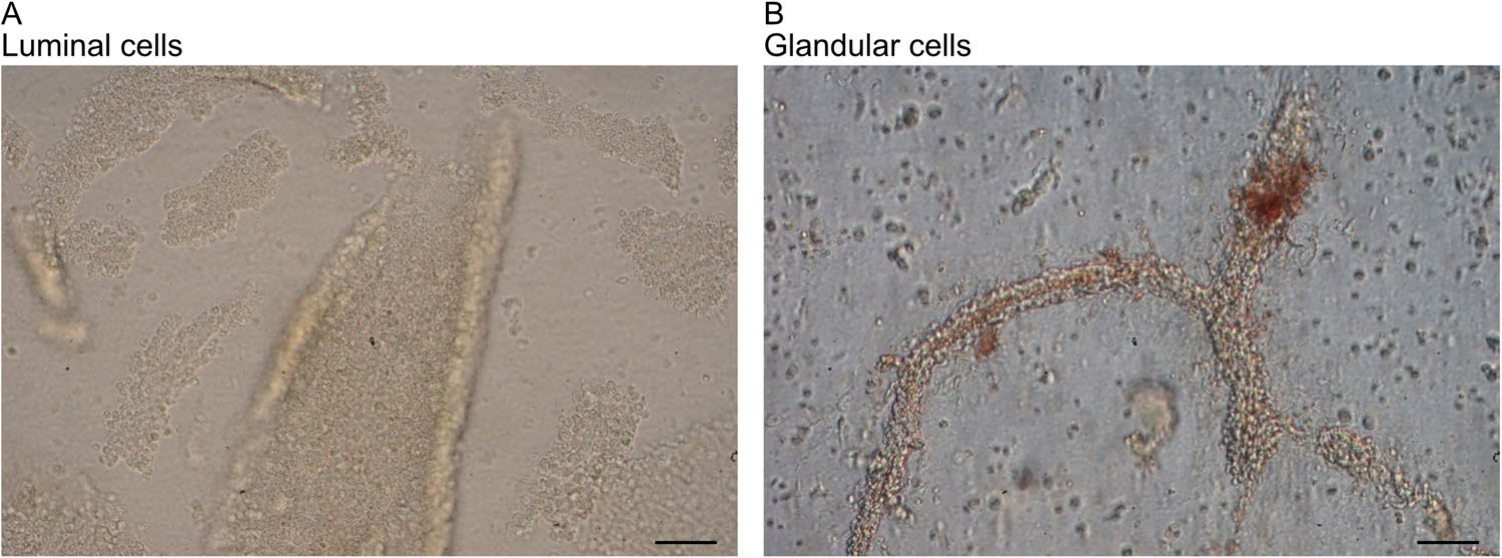
Luminal and glandular cell clusters during cell isolation. (**A**) Representative picture of PEEC-L displaying planar structure during cell isolation before the last enzymatic digestion step, scale bar = 100 µm. (**B**) Representative picture of PEEC-G displaying tubular structure during cell isolation before the last enzymatic digestion step, scale bar = 100 µm

During isolation, the enrichment of either PEEC-L or -G cells was controlled visually and was facilitated by the different shapes of the obtained cell clusters (Fig. 3). PEEC-L clusters displayed a plane sheet structure, while PEEC-G were isolated from tubular structures. The applied isolation method yielded similar cell viability (luminal: 88.6 ± 4.5%, glandular: 86.7 ± 4.5%) and number of isolated cells (luminal: 9.96 ± 1.75 * 10^6^, glandular: 8.45 ± 2.54 * 10^6^) for both cell types.

### Morphological Comparison of ALI-PEEC-L and ALI-PEEC-G

**Fig. 4:**
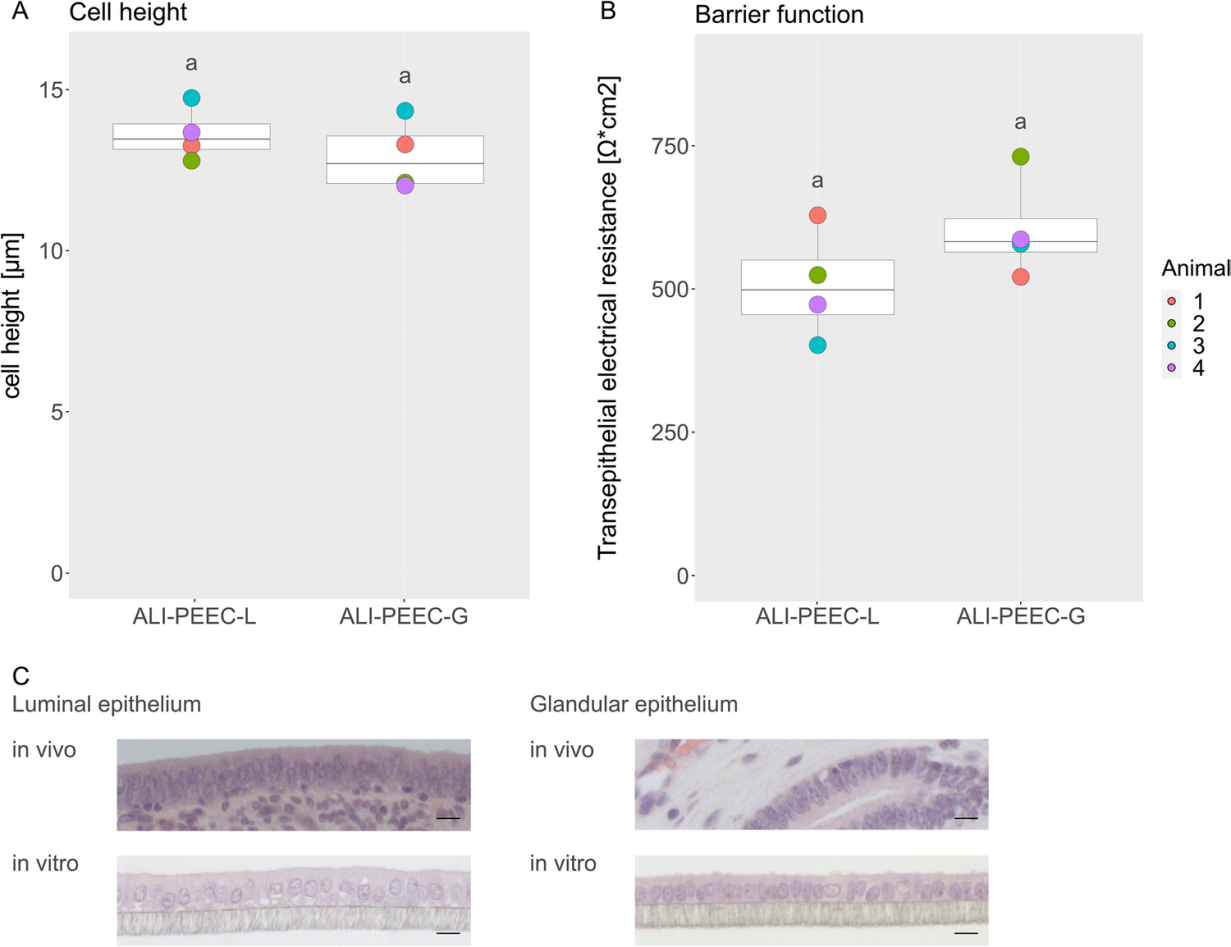
Morphology and barrier function of ALI-PEEC-L and -G after long term culture. (**A**) Cell height of PEEC-L and -G after 3 weeks at the ALI. (**B**) TEER of PEEC-L and -G after 3 weeks at the ALI. (**A**, **B**) The data are presented as a boxplot. Significant differences (*p* ≤ 0.05) between ALI-PEEC-L and -G are indicated by different superscript letters. (**C**) Representative pictures of PEEC-L and -G in vivo and in vitro. HE staining, magnification × 40, scale bar = 10 µm, *n* = 4. TEER, transepithelial electrical resistance; ALI, air–liquid interface; PEEC-L, porcine endometrial epithelial cells luminal; PEEC-G, porcine endometrial epithelial cells glandular; HE, hematoxylin–eosin

Samples for morphological analysis were taken after 3 weeks of culture (Fig. 4). ALI-PEEC-L reached a cell height of 13.62 ± 0.36 µm (mean ± SEM) and ALI-PEEC-G a cell height of 12.94 ± 0.48 µm (mean ± SEM). ALI-PEEC-L had a TEER of 507 ± 41 (mean ± SEM) after 3 weeks of culture and ALI-PEEC-G had a TEER of 604 ± 39 (mean ± SEM). The differences between ALI-PEEC-L and ALI-PEEC-G were not significant. Both cell types displayed a basolateral polarization and columnar shaped cells similar to *in vivo* morphology (Fig. 4C).

### mRNA Expression of PEEC-L and PEEC-G at Isolation and after ALI Culture

**Fig. 5:**
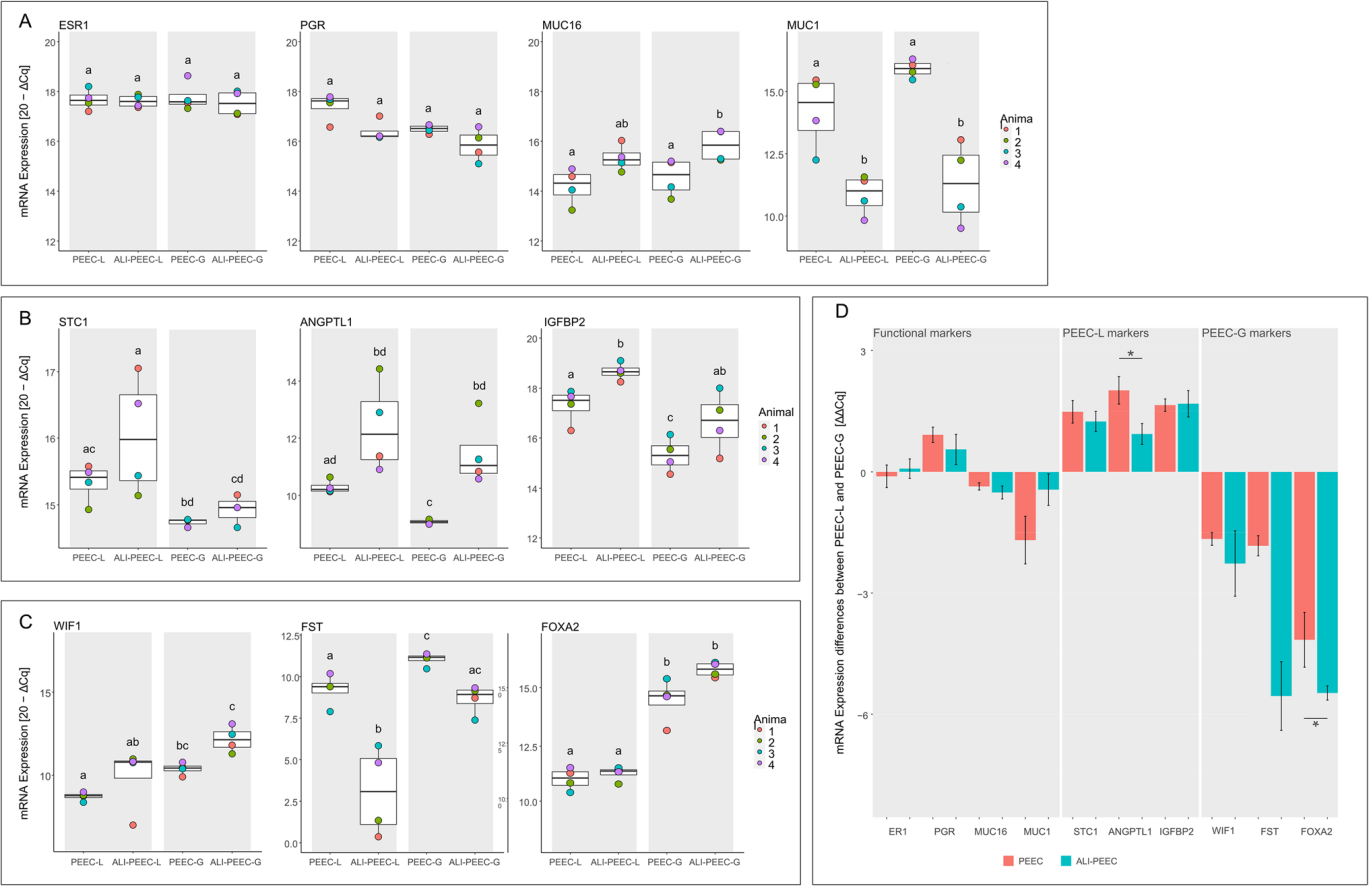
mRNA expression of PEEC-L and PEEC-G at isolation and at the ALI. (**A**) mRNA expression of the functional endometrial epithelial markers Estrogen receptor 1 (*ESR1*), Progesterone receptor (*PGR*), Mucin 16 (*MUC16*), Mucin 1 (*MUC1*) at isolation and at the ALI. (**B**) mRNA expression of the luminal epithelial markers Stanniocalcin 1 (*STC1*), Angiopoietin-related protein 1 (*ANGPTL1*) and Insulin-like growth factor-binding protein 2 (*IGFBP2*) at isolation and at the ALI. (**C**) mRNA expression of the glandular epithelial markers Wnt inhibitory factor 1 (*WIF1*), Follistatin (*FST*) and Forkhead box A2 (*FOXA2*) at isolation and at the ALI. (**A**, **B**, **C**) The results are presented as a boxplot. A high ΔCq represents a high transcript abundance. Significant differences (*p* ≤ 0.05) between PEEC-L and -G at isolation and at the ALI are indicated by different superscript letters. (**D**) mRNA expression pattern of *ESR1, PGR, MUC16, MUC1, STC1, ANGPTL1, IGFBP2, WIF1, FST* and *FOXA2* at isolation (PEEC) and after 3 weeks in culture (ALI-PEEC). The results are presented as mean delta delta quantitative cycle (ΔΔCq) ± SEM. ΔΔCq > 0 indicates higher transcript abundance in PEEC-L than in PEEC-G. Significant differences (*p* ≤ 0.05) between PEEC and ALI-PEEC are indicated by an asterisk, *n* = 4. ALI, air–liquid interface; PEEC-L, porcine endometrial epithelial cells luminal; PEEC-G, porcine endometrial epithelial cells glandular

Marker gene expression of freshly isolated PEEC-L and -G and ALI-PEEC-L and -G after 3 weeks of culture was compared. At isolation and after 3 weeks at the ALI the steroid hormone receptors *ESR1* and *PGR* did not show any difference in expression between the cell types or time points (Fig. 5A). *MUC16* was significantly higher expressed in ALI-PEEC-G, but not in ALI-PEEC-L, compared to freshly isolated PEEC-L or -G. *MUC1*, on the other hand, was significantly lower expressed at the ALI, but there was no difference between the cell types.

The luminal epithelial marker *STC1* was significantly higher expressed in PEEC-L compared to PEEC-G isolation and at the ALI (Fig. 5B). *ANGPTL1* was significantly higher expressed in PEEC-L compared to PEEC-G at isolation, but not at the ALI. In PEEC-L and -G *ANGPTL1* was significantly higher expressed at the ALI compared to isolation. *IGFBP2* showed a similar expression pattern as *ANGPTL1*. *IGFBP2* was higher expressed in PEEC-L compared to PEEC-G and was higher expressed at the ALI in both cell types compared to freshly isolated cells.

The glandular epithelial marker *WIF1* was significantly higher expressed in PEEC-G and ALI-PEEC-G compared to PEEC-L and ALI-PEEC-L, respectively (Fig. 5C). *FST* was also significantly higher expressed in PEEC-G at isolation and at the ALI. However, the expression of *FST* decreased in PEEC-L and -G at the ALI compared to freshly isolated PEEC-L and -G, while *WIF1* was by trend higher expressed at the ALI. *FOXA2* was significantly higher expressed in in PEEC-G and ALI-PEEC-G compared to PEEC-L and ALI-PEEC-L. *FOXA2* expression was maintained at the ALI in both cell types.

Comparing the mRNA expression pattern between isolation and after ALI culture by subtracting mRNA expression level (∆Cq) of PEEC-G from PEEC-L revealed a highly analogous pattern across all investigated genes (Fig. 5D). Only the subtracted mRNA expression levels of *FST* and *FOXA2* differ significantly between isolation and after ALI culture. The difference in *FST* expression was significantly lower at the ALI, while the difference in *FOXA2* expression increased at the ALI.

In summary, PEEC-L and -G showed differences in the expression of cell type specific markers at isolation indicating that an enrichment of the respective cell type was achieved. Furthermore, PEEC-L and -G maintained the overall expression pattern of functional and cell type specific markers at the ALI.

## Discussion

New *in vitro* tools are indispensable to study cell and tissue physiology including embryo-maternal communication in a reproducible and controlled manner. The here presented ALI cell culture system opens new possibilities to investigate the two endometrial epithelial cell types and their unique functions separately.

In a first aspiration to differentiate and maintain PEEC at the ALI, only LE cells were cultured. After three weeks of culture, these PEEC-L formed baso-apical polarized cells growing in monolayers on the hanging insert. Subsequently, PEEC-L were cultured in 3 additional media. The tested media differed in the supplementation of growth factors and FBS. In general, serum-free medium has the advantage of being chemically defined. The application of FBS in cell culture medium is controversial due to issues regarding quality and reproducibility [36]. Primary epithelial cells originating from the porcine oviduct were reported to form differentiated epithelial monolayers when cultured in SF medium at the ALI [23, 37]. In PEEC-L, however, SF medium did not foster appropriate differentiation; the cells stayed cuboidal and did not form columnar shaped monolayers. Similar to porcine oviduct epithelial cells grown at the ALI, epithelial height was inversely correlated with TEER, and *in vivo*-like morphology corresponded to moderate TEER values [17]. Even the simple one-step medium approach led to highly differentiated ALI-PEEC-L monolayers and is therefore applicable for ALI culture of this cell type. In order to apply a medium with as defined properties as possible, the two-step protocol with P medium for the submerged pre-culture and NU medium of the ALI culture was chosen to conduct all further experiments.

In the second experiment, both PEEC-L and PEEC-G were isolated from the same uteri. Separation of glandular and luminal cells was ensured by isolation from different localizations within the tissue, microscopic inspection of the shape of isolated structures before dissociation into single cells, and subsequent gene expression analysis. Both cell types reproducibly formed polarized monolayers with a similar *in vivo*-like cell height and TEER after 3 weeks at the ALI [17, 38–40]. However, absolute numbers of the LE and GE cell layer height *in vivo* differed between studies [39–41] and comparison of *in vivo* and ALI-PEEC cell height is generally delicate, as cell height depends on sample processing, fixation and dehydration procedures which are not identical between studies and are also divergent for *in vivo* samples and *in vitro* samples from ALI cultures [42].

Overall, the gene expression differences of PEEC-L and -G observed at isolation were maintained in ALI-PEEC-L and ALI-PEEC-G, respectively. The hormone receptors *ESR1* and *PGR* did not show any difference in expression between cell types or time points. *In vivo* both receptors are expressed in LE and GE and are essential for appropriate endometrial response to estradiol-17β and progesterone [33, 34]. In the present study, only non-cyclic peri-pubertal donors were included and the ALI-PEEC were neither supplemented with estradiol-17β nor with progesterone. The stable expression of both receptors suggests that the PEEC-L and -G maintained their hormone responsiveness at the ALI.

*MUC16* expression was either stable or increased after ALI culture compared to PEEC- L or -G at isolation. Interestingly, *MUC1* was downregulated at the ALI. *In vivo MUC1* expression has been shown to be upregulated by progesterone and locally downregulated in the presence of a conceptus to enable attachment [43, 44]. Conversely, *MUC16* was demonstrated to be downregulated in the luteal phase in humans and bovine [45, 46]. To verify *in vivo*-like *MUC1*, *MUC16,* but also *ESR1* and *PGR* expression of PEEC-L and -G at the ALI, an estrous cycle simulation or the co-culture with embryos should be conducted [25]. *MUC16* is necessary for the mucosal epithelium to maintain its barrier function, while a *MUC1* is insignificant for barrier function [47]. Besides TEER and morphology, the stable expression of *ESR1, PGR* and *MUC16* highlights that PEEC-L and -G maintained characteristics of the functional porcine endometrial epithelium at the ALI.

*STC1*, *ANGPTL1* and *IGFBP2* were applied as luminal [27, 28, 31] and *FST*, *WIF1* and *FOXA2* as glandular markers [8, 29, 30]. *FOXA2* is a marker exclusively expressed in glandular cells in the endometrium across several species. The other selected markers are not strictly attributable to one of the cell types, but higher expression levels were reported for either PEEC-L or -G. At isolation, the markers were higher expressed in the respective cell types, indicating that during isolation both cell types were mainly separated from each other. After 3 weeks of culture at the ALI in PEEC-L, only *STC1* was still significantly higher expressed. However, the levels of LE marker gene expression were maintained at the ALI in PEEC-L.

All glandular markers were significantly higher expressed in PEEC-G compared to PEEC-L both at isolation and after ALI culture. The expression of FOXA2 in both cell types indicates that some PEEC-G were isolated together with PEEC-L [30]. Possibly, *FOXA2* positive cells located at the top of the glandular invagination detached from the endometrium together with the PEEC-L during the mechanical removal. However, as *FOXA2* is significantly higher expressed in PEEC-G at isolation and after ALI culture compared to PEEC-L an enrichment of the respective cell type was achieved. Further, *WIF1* and *FOXA2* expression level was maintained at the ALI. On the other hand, *FST* was lower expressed at the ALI than at isolation. The loss of significant differences in marker gene expression between the cell types and down regulation of marker genes at the ALI, respectively, might point to a de-differentiation of the cells due to the missing 3D-structure of the ALI-culture system, the likewise missing contact with stromal cells or a lack of hormonal stimulation [48]. To provide a more *in vivo*-like sub-epithelial microenvironment, ALI-PEEC-L and -G could be cultured together with stromal cells in recently made commercially available scaffolds, which would result in a segmented tissue-like cell culture. [49].

### Limitations of the present study

A major limitation is the lack of an experimental proof for the hormone responsiveness of the cell cultures. Therefore, stimulation experiments with estradiol-17β and progesterone are indicated. A second limitation is the incomplete separation of cell types during the isolation process, as indicated by the detection of FOXA2 expression in the luminal epithelial cells. Thus, an enrichment rather than an absolute separation of each cell type was achieved.

## Conclusion

We conclude that PEEC-L and -G formed differentiated epithelia by 3 weeks of culture at the ALI as illustrated by morphological analysis and TEER. They furthermore maintained cell type-specific characteristics shown by the stable expression of marker genes over the culture period. We thus here present a reproducible ALI cell culture system of both luminal and glandular epithelial cells of the porcine endometrium. In the future, this cell culture system could be used for toxicological or endocrinological studies as well as for the study of host–pathogen and embryo-maternal interactions *in vitro*.

## Data Availability

All data generated or analyzed during this study are included in this published article.
